# Cancer Vaccines: Toward the Next Breakthrough in Cancer Immunotherapy

**DOI:** 10.1155/2020/5825401

**Published:** 2020-11-17

**Authors:** Yuka Igarashi, Tetsuro Sasada

**Affiliations:** ^1^Kanagawa Cancer Center, Research Institute, Division of Cancer Immunotherapy, Japan; ^2^Kanagawa Cancer Center, Cancer Vaccine and Immunotherapy Center, Japan

## Abstract

Until now, three types of well-recognized cancer treatments have been developed, i.e., surgery, chemotherapy, and radiotherapy; these either remove or directly attack the cancer cells. These treatments can cure cancer at earlier stages but are frequently ineffective for treating cancer in the advanced or recurrent stages. Basic and clinical research on the tumor microenvironment, which consists of cancerous, stromal, and immune cells, demonstrates the critical role of antitumor immunity in cancer development and progression. Cancer immunotherapies have been proposed as the fourth cancer treatment. In particular, clinical application of immune checkpoint inhibitors, such as anti-CTLA-4 and anti-PD-1/PD-L1 antibodies, in various cancer types represents a major breakthrough in cancer therapy. Nevertheless, accumulating data regarding immune checkpoint inhibitors demonstrate that these are not always effective but are instead only effective in limited cancer populations. Indeed, several issues remain to be solved to improve their clinical efficacy; these include low cancer cell antigenicity and poor infiltration and/or accumulation of immune cells in the cancer microenvironment. Therefore, to accelerate the further development of cancer immunotherapies, more studies are necessary. In this review, we will summarize the current status of cancer immunotherapies, especially cancer vaccines, and discuss the potential problems and solutions for the next breakthrough in cancer immunotherapy.

## 1. Introduction

When one hears the word “vaccine,” many people think of vaccines against infectious agents, such as viruses and bacteria. For many years, such vaccines have protected humankind from catastrophic infections [1]. The mechanism through which vaccines provide protection against an infection involves the artificial induction of immune responses against infectious antigens by inoculating a healthy person with attenuated/detoxified bacteria, viruses, or extracted toxins [2]. The aim of a vaccine is to prevent or reduce the severity of life-threatening infectious diseases (prophylactic vaccines). Acquisition of immune memory from vaccines is often effective over long periods of time [3]. A global system of routine immunization against highly prevalent infections has been effectively established; this has resulted in multiple individuals developing immunity against various diseases. Additionally, the World Health Organization (WHO) recommends the administration of free mumps and varicella vaccines in developed countries, where vaccines are recognized as being among the most versatile and important preventive measures [4].

The immune system is directed at maintaining homeostasis in living organisms by monitoring the invasion of foreign pathogens (and associated factors), as well as the presence of abnormal or transformed cells, for their exclusion. This process is called immune surveillance [5]. In daily life, humans are exposed to external factors, such as bacteria, viruses, or harmful substances. Additionally, humans are exposed to various factors that lead to abnormalities and transformations in normal cells. However, it is rare that these exposures or transformations immediately lead to the development of disease, because humans are strongly protected by their immune systems. When there is an imbalance between extraneous stimuli and biological defense and when components of the immune system are unable to eliminate the pathogen or malfunctioning cells, conditions, such as infections and cancers, develop [6].

## 2. History of Cancer Vaccines

**Figure 1:**
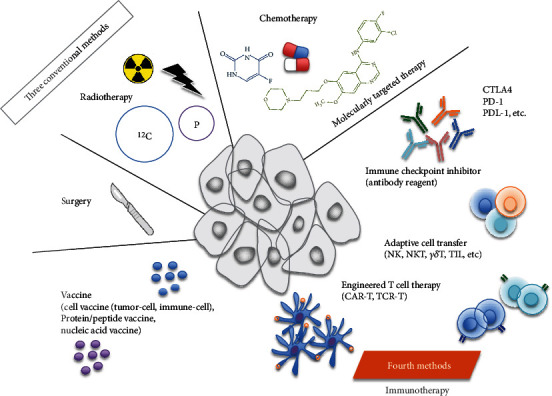
Cancer treatment methods. Conventional methods for cancer treatment include surgery, chemotherapy, and radiation therapy, which remove or directly attack the cancer cells. Recent advances in medical science have resulted in the addition of cancer immunotherapies as a fourth treatment method, which can indirectly attack cancers by regulating the patient's immunity.

Till now, chemotherapy, radiotherapy, and surgical excision are the three major cancer treatment methods that directly remove or target the cancer cells. In addition, immunotherapies for the treatment of cancer by leveraging the innate and/or adaptive immune function in humans have been extensively studied. The critical role of the tumor microenvironment (TME), which consists of cancer, stromal, and immune cells that interact with each other, is becoming increasingly apparent. Therefore, cancer immunotherapies have been reconsidered and recognized as the fourth treatment method (Figure 1) [7–9]. Preventive and therapeutic vaccines exist as representative strategies for cancer immunotherapy. The former is aimed at inducing immune memory by administering vaccines to healthy persons to prevent morbidity due to a particular cancer. The latter is administered to patients with cancer for disease management by reinforcing or reactivating the patient's own immune system.

Clinical application of cancer vaccines has faced extreme hurdles despite multiyear research and development efforts by many researchers. The administration of streptococcal organisms (Coley's toxin) as a therapeutic vaccine in patients with sarcoma in the 1890s by Dr. William B. Coley was the first report of cancer immunotherapy [10]. In this strategy, for both prophylactic and therapeutic purposes, specific immune responses were induced against certain sarcoma antigens. The development of this cancer vaccine was based on the clinical findings that the incidence of cancer was low in patients with certain infectious diseases. This phenomenon may reflect the fact that infection and inflammation induce the exposure of antigens abnormally expressed by cancer cells. It might also be a secondary effect, where the immunological memory acquired from past infection or inflammation affects the cancer cells. Similarly, antibodies against abnormal cell surface-associated mucin (MUC1) produced during mumps infection decreases the incidence of ovarian cancer [11]. Moreover, Bacillus Calmette-Guerin has been used as a tuberculosis vaccine for a long time [12, 13] and is now also widely employed as a therapeutic vaccine against bladder cancer.

As some types of cancers are caused by infectious viruses, prophylactic vaccines against viral infection can prevent cancer development [14, 15]. The Food and Drug Administration (FDA) has approved two types of prophylactic cancer vaccines for targeting the human papillomavirus (HPV) and hepatitis B virus (HBV) to prevent HPV-related cancers and HBV-related hepatocarcinoma. However, only a few types of cancer are caused by viral infections. In addition, the global vaccination rate for these prophylactic vaccines is not high. Thus, the number of patients in whom the antiviral vaccines have successfully prevented cancers is limited.

## 3. Immunological Characteristics of Cancers

Progress in the latter half of the 20^th^ century in tumor immunology and molecular biology has been remarkable. Numerous studies have vigorously investigated the mechanism by which tumors evade the immune system. These efforts have identified a mechanism called “cancer immunoediting” as one of the immune evasion tactics utilized by the tumors [16]. Cancer immunoediting appears to be the consequence of antitumor immune responses mediated by antigen recognition in the tumor environment. The interaction between the immune system and cancer cells, which originally contained specific genetic mutations, may cause a selective and biased proliferation of the clones that have lost these mutations, leading to tumor escape from the immune system. For these cancers, the immune system might not discriminate cancer cells that have lost specific antigens from normal host cells, resulting in the possibility that cancer cells do not elicit a strong exclusionary immune response.

Additionally, the presentation of cancer cell antigens to the T cells differs from the presentation of antigens by mature antigen-presenting cells (APCs) in the context of the participation of costimulatory molecules. Antigen presentation by the APCs involves the presence of a simultaneous second signal from costimulatory molecules, such as CD28, for inducing T cell activation during antigen recognition. This second signal controls the subsequent T cell response [17]. When the antigen is presented to T cells without the second signal, antigen stimulation itself might be ignored; this is termed unresponsive anergy resulting in the loss of the antigen-specific T cell responses. Because cancer cells lack such critical second signals, they may not efficiently induce T cell responses, even if strong cancer-specific antigens derived from genetic mutations are presented to T cells.

Immunosuppressive cells (e.g., myeloid-derived suppressor cells (MDSCs) and regulatory T (Treg) cells) are recruited to the TME by chemotactic factors derived from tumor, stromal, or other immune cells and convey negative signals to the antitumor immune cells via the expression of inhibitory ligands (e.g., programmed death-ligand 1 (PD-L1)) and the secretion of immunosuppressive factors (e.g., interleukin 10 (IL-10), transforming growth factor-beta (TGF-b), and prostaglandin E2 (PGE 2)). In doing so, an environment favoring cancer cell growth is created [18].

## 4. Current Status of Cancer Vaccines

The identification of the mechanisms used by the cancer cells to evade the immune system has resulted in the development of several tools including antibodies, peptides, proteins, nucleic acids, and immunocompetent cells (dendritic cells, T cells, etc.) for cancer immunotherapy. With respect to cancer vaccines, these techniques fall into three major categories based on format and content, i.e., cell vaccines (tumor or immune cells), protein/peptide vaccines, and nucleic acid vaccines (DNA, RNA, or viral vector).

### 4.1. Cell Vaccines (Tumor Cell Vaccines or Dendritic Cell (DC) Vaccines)

An autologous tumor cell vaccine using a patient's own cancer cells is one of the vaccine strategies being evaluated. In this approach, irradiated tumor cells are administered along with an adjuvant. As this vaccine uses tumor cells, it might be possible to induce T cells specific to any antigen expressed by the used cells. However, the limitation of this strategy is that a sufficient number of cells is sometimes difficult to obtain [19–22]. This approach has been attempted in many tumors, including lung cancer [22–24], colorectal cancer [20, 25–27], melanoma [28–30], renal cell carcinoma [31–33], and prostate cancer [19, 34]. In many cases, tumor cells are genetically modified to add functions, such as cytokine production (e.g., IL-2 [35] and granulocyte-macrophage colony-stimulating factor (GM-CSF) [36–39]) and costimulation (e.g., B7-1) [32]. GVAX is a cancer vaccine based on tumor cells genetically modified to secrete GM-CSF. It is used after cancer irradiation to stop the uncontrollable growth of cancer cells. There are two types of GVAX vaccine approaches, one using autologous cells (patient-specific), and the other using allogeneic cells (non-patient-specific). GVAX phase 1/2 clinical trials in patients with non-small-cell lung carcinoma have shown good results, correlating GM-CSF secretion and patient prognosis [40]. However, no effects have been seen in phase 3 clinical trials for prostate cancer [41]. Currently, several phase 2 trials of GVAX therapy for advanced pancreatic cancer have been conducted in combination with body radiation or mesothelin-expressing *Listeria monocytogenes* vaccine or cyclophosphamide (CY), with promising results.

An allogeneic tumor cell vaccine that includes tumor cell lines, such as Canvaxin [42], may overcome the limitation associated with the individualization of autologous tumor vaccines. These vaccines have been studied in prostate [43, 44], breast [45], and pancreatic cancers [46]. Although homologous GVAX against metastatic castration-resistant prostate cancer (CRPC) did not achieve its phase 3 clinical trial goals, a combination strategy using allogeneic GVAX against CRPC and an immune checkpoint inhibitor is being studied [47, 48].

A new therapeutic approach focuses on DCs that present antigens to T cells and promote immune system activation. DC therapy has been intensively studied since the late 1990s [49], when Dr. Ralph M. Steinman, who discovered DCs, recognized their potential, and the possibility of using DCs as a vaccine [50]. A variety of antigens, including tumor cells, tumor-derived proteins or peptides, and DNA/RNA/virus, could be potentially loaded on DCs. There are additional methods, such as the fusion of DCs with tumor cells. Several receptor types are expressed on the surface of DCs. For example, binding of an antigen to a lectin-like receptor known as scavenger receptor on DCs is reported to induce antigen-specific suppressive CD4(+) T cells. It is noteworthy that not all antigen presentation by DCs contributes to immune activation [51].

In 2010, Provenge (sipuleucel-T; Dendreon Corporation) was approved by the FDA as a prostate cancer vaccine and has drawn attention to the use of autologous immune cells for immunotherapy. It is a crude leukocyte fraction recovered from the peripheral blood of an individual patient, which is then cultured with a prostate carcinoma antigen (prostatic acid phosphatase (PAP)) in the presence of GM-CSF. DCs are the main active components of Provenge (about 11.2% [52]) and display the PAP antigen to artificially stimulate and induce antigen-specific T cells in patients. Provenge is a good example of the complexity of personalized medicine, as personalized cancer vaccines can be effectively created using this approach. Nevertheless, all of the processes involved in the production of a personalized vaccine, from sample collection to transporting, processing, shipping, and administration of the cells, need to be customized for each patient, leading to increased labor and cost.

### 4.2. Protein/Peptide Vaccines

Protein/peptide vaccines can induce immunity against specific antigenic epitopes derived from the vaccinated protein/peptides that are expressed in cancer cells (and preferably not expressed in normal tissues). When an artificially synthesized antigen protein/peptide is administered, it is taken up by professional APCs and presented in complex with the HLA molecules on the cell surface. When T cells recognize the antigens, cancer-specific immune responses are induced. Antigenic epitopes derived from tumor-associated antigens (TAAs) capable of binding HLA have been extensively identified. In addition, antigens derived from cancer-specific gene mutations that are not present in normal tissues have recently attracted attention as neoantigens. To efficiently search for these neoantigens, algorithm-based computer searches are rapidly being developed [53–55].

As many early protein/peptide vaccine clinical trials have resulted in favorable results, phase 3 trials have been conducted to confirm the results. Unfortunately, most of these trials have failed, suggesting that single-protein/peptide vaccines do not exert sufficient antitumor effects [56]. Even if T cell responses were induced by protein-/peptide-derived epitopes, the antitumor effects can rarely be achieved with low response rate (less than 10%) [57, 58]. These unexpected results may be explained by several factors, including tumor immune escape mechanisms and immunosuppressive TMEs [59].

There may be problems with the vaccine formulations; most peptide vaccines developed thus far consist of short-chain peptides (SPs) restricted to MHC class I. Unlike long-chain peptides (LPs), SPs are able to bind to any cells without processing and might induce anergy if presented to CD8 T cells without the secondary costimulatory signal [60, 61]. In these situations, immune tolerance is induced, creating an environment favorable for cancer progression. Furthermore, MHC class I-restricted SPs cannot contribute to the activation of MHC class II-restricted helper T cells, which are important for efficient cytotoxic T lymphocyte (CTL) induction. However, LPs can be processed to both MHC class I- or class II-restricted antigens and presented by professional APCs, but not by other cell types. Professional APCs that present LP-derived antigens can activate CTL or helper T cells without inducing anergy by transmitting signals via both T cell receptors (TCRs) and costimulatory molecules [60, 62–64]. Currently, the development of LP vaccines that contain epitopes for both CTL and helper T cells is being actively pursued. In addition, a novel method with an immunostimulatory adjuvant has been developed to improve the responsiveness to peptide vaccines [65–68].

### 4.3. Nucleic Acid Vaccines (DNA, RNA, or Viral Vector)

Nucleic acid (DNA/RNA) vaccines have advantages in that they can simultaneously activate immunity against multiple epitopes [69]. Further, these vaccines are inexpensive and can be synthesized stably. When an immunogenic viral vector is used, the adjuvants are not as important, unlike in peptide vaccines. However, when a viral vector is not used, developing an efficient delivery method becomes an important issue, especially as the efficiency of nucleic acid uptake into cells might be low [70].

DNA vaccines have shown promise in several preliminary studies [71, 72]. For example, VGX3100, a DNA vaccine for cervical cancer, is in phase 3 clinical trials (NCT03185013) [73]. RNA vaccines, unlike DNA vaccines, are not incorporated into the genome, thereby preventing carcinogenicity. Additionally, unlike DNA vaccines that need to enter the nucleus, RNA vaccines can function in the cytoplasm. Therefore, clearance is quick and the possibility of causing side effects might be low. RNA is more easily degraded than DNA, but stability can be enhanced by various modifications, such as formulations with liposomes or stabilizing adjuvants [74–77]. Techniques have also been developed to stabilize the RNA molecule itself (5′ cap structure, untranslated regions, and codon usage in translated regions) [78]. Phase 1/2 studies are ongoing for melanoma and kidney cancer [79–81]. A phase I study of liposome-encapsulated mRNA for patients with advanced melanoma is also underway [82]. Further development of nucleic acid delivery methods will serve as a breakthrough in nucleic acid vaccines.

Viral vectors are used to efficiently carry nucleic acids for vaccines. Adjuvants are not required for viral vectors, which can activate innate immunity and also induce immune responses to viruses. Commonly used viral vectors are derived from poxvirus, vaccinia virus, adenovirus, and alphavirus and are attenuated or replication-defective for safety. For example, some modified vaccinia virus Ankara (MVA) vector-based vaccines are used to target the renal cell carcinoma 5T4 and MUC1 antigens [83, 84]. Recombinant adenoviruses are commonly used in cancer gene therapy because they can transduce dividing and nondividing cells and are easy to produce ([85–87], NCT00583024, NCT00197522). Herpes simplex virus type 1 (HSV-1), an enveloped dsDNA virus, is used as an oncolytic virus. HSV-1 expressing GM-CSF (e.g., OncoVEXGM-CSF) is useful in melanoma [88, 89]. The disadvantage of viral vectors is that repeated administration might be difficult due to the induction of antiviral immune responses. For this reason, a heterologous prime-boost strategy is developing. For example, PROSTVAC, a vaccine consisting of two poxvirus vectors that express tumor-associated antigen prostate-specific antigen (PSA) combined with 3 immune-enhancing costimulatory molecules collectively designated as TRICOM (LFA-3, ICAM-1, and B7.1), is under development by Bavarian Nordic (Denmark) to stimulate an immune response in prostate cancer [90–92]. The results of the PROSTVAC-VF/TRICOM phase 3 trial were not as expected, but this vaccine shows promise when combined with immune checkpoint inhibitors (NCT02933255, NCT02506114). In addition to viruses, bacteria and yeasts are also attracting attention as new vaccine carriers [93, 94].

### 4.4. Combination Therapy with Cancer Vaccines

Until recently, monotherapies using cancer vaccines often had minimal clinical effects except for certain specific cancer types. The relatively low efficacy of monotherapies was attributed to the multifaceted immune evasion mechanisms of cancer, which are difficult to control by either cancer vaccine alone. As described earlier, the immunosuppressive TME [95] may override any antitumor effects elicited by the cancer vaccine.

In accordance with the development of various immunotherapy types, more attention has focused on combination therapies. Several different approaches, including conventional chemotherapy/radiation therapy or the latest antibody therapies [96, 97], have been attempted in combination either simultaneously or in sequence with immunotherapies.

### 4.5. Issues in the Clinical Development of Cancer Vaccines

During the development of many conventional cancer vaccines for clinical use, the test designs may have been flawed. For example, clinical effects of cancer vaccines were often evaluated in patients with a terminal diagnosis whose immune conditions were already substantially compromised by exposure to several other treatments, such as surgery and chemotherapy/radiotherapy, or by progression of the disease.

In addition, it is essential to develop accompanying technologies such as adjuvants, manufacturing, and delivery methods to employ vaccines and to obtain expected results in clinical settings [98, 99]. The introduction of such state-of-the-art technology may create additional hurdles due to current drug regulations, which are controlled by regulatory authorities in individual countries, including the FDA, European Medicines Agency (EMA), and Ministry of Health, Welfare, and Labour in Japan [100, 101]. These hurdles are especially important in the development of drugs related to cancer immunotherapy, where it can be difficult to set up a primary endpoint to evaluate the effectiveness of a therapy. Thus far, some cytotoxic drugs under investigation have been known to generally prolong disease-free survival (DFS), but not overall survival (OS), but drugs that extend OS without improving DFS are not very common. Nevertheless, regarding immunotherapy approaches, a situation often arises where OS is extended even if tumor reduction is not observed [102]. In addition, as the immune status of individual patients may be affected by various factors such as age and past treatment history, it is difficult to adequately predict the antitumor effects in nonclinical studies.

## 5. The Current Status of Other Cancer Immunotherapies

In addition to cancer vaccines, other types of cancer immunotherapies are in development. These have been described below.

### 5.1. Tumor-Infiltrating Lymphocyte (TIL) Therapy

When tumor tissues are available, TIL therapy is a promising approach. For TIL therapy, T cells that recognize cancer-specific antigens are collected from tumor tissues in patients with cancer, artificially reactivated by using T cell-stimulating agents, such as a high IL-2 concentration, and are then returned to the patients. This approach is potentially simple because genetic modifications are not required, but the clinical effects might be dependent on the amount and quality of infiltrating lymphocytes collected from tumor tissues. Dudley et al. [103] have reported much information on TIL therapy in patients with cancer. Many researchers and doctors are fascinated by their reports on the potential of TIL therapy in melanoma [104–106]. In the latest method, IL-2 and TILs are simultaneously administered to patients to enhance clinical effects [107–109]. In addition, the same group has conducted similar TIL studies for advanced cervical cancer with potential success [110].

### 5.2. TCR/CAR-T Cell Therapy

The availability and performance of TIL therapy might be dependent on whether sufficient numbers of high-quality antigen-responsive T cells can be secured from tumor tissues in individual patients. To circumvent such limitations, peripheral blood mononuclear cell- (PBMC-) derived lymphocytes, which artificially express a desired TCR or chimeric antigen receptor (CAR), have been devised and clinically applied as a novel T cell therapy. TCR-T therapy is a therapeutic method using T cells transduced with antigen-specific TCRs [111]. CAR-T therapy is a method of administering T cells with a CAR gene, which is composed of a fragment derived from a cancer antigen-recognizing antibody gene, gene fragments from intracellular TCR domains, and other T cell costimulatory molecules.

CAR-T therapy is extremely effective in blood cancers [112]. In 2017, the FDA approved CD19 CAR-T therapy for B cell acute lymphoblastic leukemia (ALL) which has become refractory to first-line treatment or has recurred more than once [113, 114]. Although CAR-T therapy is highly effective, the issues surrounding the cost of care with CAR-T therapy (more than $500,000 for one administration) have yet to be resolved. Notably, CAR-T is effective when a tumor antigen is monolithic or tumor tissue heterogeneity is low (e.g., a genetically uniform tumor, which is often the case with blood tumors). However, the efficacy of CAR-T therapy to solid tumors remains limited, because these tumors generally show more heterogeneity [115].

Another problem with TCR/CAR-T cell therapy in solid tumors is the suppressive TME, which inhibits effective infiltration and/or accumulation of administered T cells inside the tumors. Therefore, a method for effectively driving infiltration of the genetically modified cells into the TME remains out of reach. Moreover, since target antigens are not uniformly expressed in solid tumors and are different depending on the cancer type, stage, and patient, current TCR/CAR-T cell therapy is not widely used in patients with cancer. Furthermore, commonly available target antigens similar to CD19 in blood cancers remain to be identified in solid cancers [116].

### 5.3. Immune Checkpoint Inhibitors

In the 1990s, immune checkpoint molecules, including cytotoxic T-lymphocyte-associated protein 4 (CTLA-4) [117] and programmed death 1 (PD-1) [118], were discovered. Both molecules suppress T cell activation, which is important for exerting antitumor effects. The expression of these molecules increases in proportion to T cell activation, and they function as a defense system for organisms to inhibit excessive T cell activation and prevent autoimmune responses. The activity of T cells is suppressed by ligands binding to CTLA-4 and PD-1. For example, the costimulatory molecule CD80/CD86 expressed on APCs enhances T cell activation by simultaneously binding to CD28 on T cells during antigen presentation. In contrast, CTLA-4 is induced on activated T cells and inhibits the costimulatory signal mediated by CD80/CD86 through competition with CD28. Additionally, antigen-stimulated T cells express PD-1, which suppresses excessive T cell activation by binding to its ligand, PD-L1 or programmed death-ligand 2 (PD-L2) [119]. Additional constituents of the cancer microenvironment, such as DCs [120], macrophages [121], and fibroblasts [122], can also express PD-L1 and/or PD-L2, forming an immunosuppressive environment where cancer is more prone to progress.

In addition to evading the immune system by deleting cell surface molecules required for antigen recognition, cancer cells can often directly use (e.g., hijack) the immunosuppression system, such as immune checkpoints. Some cancer cells strongly express ligands for immune checkpoint molecules, such as PD-L1 and PD-L2 [123, 124]. A remarkable antitumor effect can thus be observed in some patients by administering treatments that block inhibitory molecules in T cells [125, 126]. Once antitumor activity is induced and immune memory is established by immune checkpoint inhibitors in patients with cancer, their clinical effects should be long-lasting.

In 2011, both the FDA and EMA approved anti-CTLA-4 antibody treatment (ipilimumab) for patients with melanoma [127, 128]. Since then, several immune checkpoint inhibitors, anti-PD-1, and anti-PD-L1 antibodies have been approved every year.  Table 1 shows several currently approved immune checkpoint inhibitors and their indications. These immune checkpoint inhibitors often show only limited and/or transient efficacy, reflecting the complexities of antitumor immunity [129, 130]. For example, immune checkpoint inhibitors are less effective in microsatellite stable tumors [131]. Such tumors often express only minor genetic abnormalities and thus possess little antigenic capacity. Therefore, these tumors fail to induce cancer antigen-specific T cells, resulting in unresponsiveness to immune checkpoint inhibitors. In addition, the immunosuppressive TME might also affect the clinical effects of immune checkpoint inhibitors.

## 6. Challenges for the Future Development of Cancer Immunotherapies: Requirement of Lymphocyte Infiltration/Accumulation within Tumors

Analysis of the interaction among tumor infiltrating immune cells (e.g., DCs, MDSCs, CD4/8T cells, and Tregs), stromal cells, and tumor cells is essential to understand the relationship between the TME and the clinical effects of cancer immunotherapies. Especially, many clinical trials using cancer immunotherapies indicate that TIL abundance in the tumor is an important prognostic factor [132]. For example, the magnitude of lymphocyte infiltration to tumors significantly contributes to immune checkpoint inhibitor effectiveness [133, 134]. The effectiveness of immune checkpoint inhibitors is high against inflammatory-type tumors (hot tumors) [135, 136], where immunocompetent cells are sufficiently present. In contrast, in immune desert-type tumors (cold tumors) with less intratumoral lymphocyte infiltration [137], immune checkpoint inhibitors are less effective.

**Figure 2:**
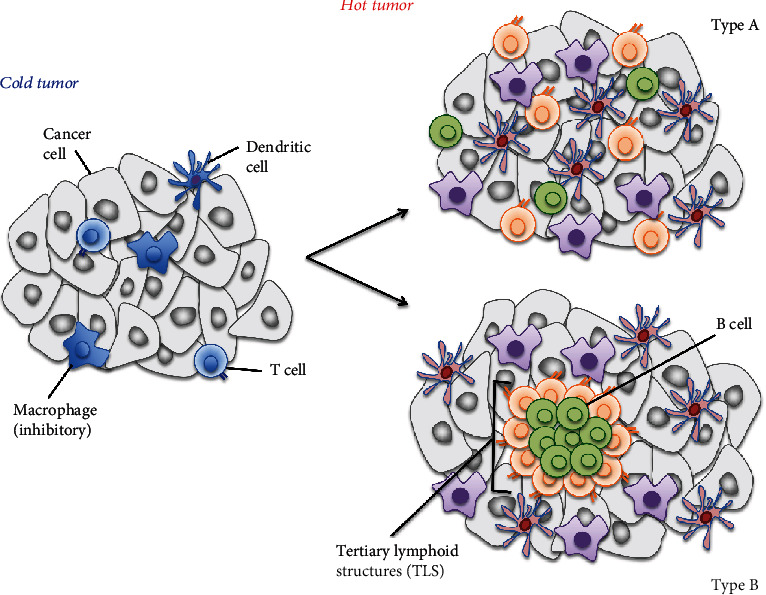
Classification of tumors by immune cell infiltration. Tumor types can be classified by the level of immune cell infiltration into tumors. “Cold tumor,” characterized by the poor infiltration of immune cells, is reported to be one of the reasons why immune checkpoint inhibitors are ineffective. Contrastingly, a “hot tumor” is characterized by the abundant infiltration of immunocompetent cells, showing good responses to immune checkpoint inhibitors. Recently, aggregated infiltration of immune cells, known as the tertiary lymphoid structure (hot tumor, type B), has gained increasing attention compared to separated infiltration of immune cells (hot tumor, type A).

The difference between these tumor types might depend on whether the immune responses to tumors are maintained or not, suggesting that the recruitment/accumulation of tumor-specific T cells is the limiting factor for therapeutic effects. Treatment could thus be optimized by the development of techniques to increase lymphocyte infiltration into tumors either before or during immunotherapy treatment (Figure 2).

For the development of more effective CAR-T cell therapies, modification with cytokine/chemokine-related genes was recently reported to efficiently drive infiltration of administered CAR-T cells into tumors [138]. Nevertheless, few studies are available on the development of methods for changing cold tumors to hot tumors. Thus, more studies are needed to develop a method to efficiently recruit/accumulate lymphocytes within tumors in combination with immunotherapeutic approaches, including cancer vaccines to stimulate antigen-specific immunocompetent cells, antigen-specific cytotoxic T cell therapies using *ex vivo* genetic modification, or blockade of inhibitory signals from the tumor.

### 6.1. Induction of Tertiary Lymphoid Structures (TLS)

As shown in “hot tumor (type B)” in Figure 2, the accumulation of various immune cell types, especially the formation of lymphoid follicles where immunocompetent cells can exchange information in the tumor, may be important [139, 140]. Indeed, lymphoid follicles in tumors, known as TLS, are associated with better prognosis in several cancers and have recently attracted attention. Several TLS-inducing factors have been reported, including CCL19, CCL21, CXCL12, CXCL13, LIGHT, and lymphotoxin [141–144]. While some basic studies for inducing lymphoid follicles have been conducted in mice, there are no promising methods that are clinically applicable for human therapy. Since efforts so far have been generally focused on a single factor, they might fail to reproduce an induction of TLS due to the complicated mechanisms within the TME. A novel method to efficiently induce TLS in humans, combined with other immunotherapies, such as cancer vaccines and immune checkpoint inhibitors, may be a promising approach for tumor control.

## 7. Conclusions

The clinical application of immune checkpoint inhibitors has greatly advanced cancer treatment. However, their effects are limited because tumor cells use various mechanisms to evade antitumor effects. To overcome these mechanisms and to improve the versatility of current cancer immunotherapies, it is necessary to understand the TME in more detail and develop novel approaches, including cancer vaccines. We hope that this review will facilitate the further development of cancer immunotherapies.

## Figures and Tables

**Table 1:** A list of currently approved cancer immunotherapies.

	FDA/EMA	MHLW (Japan)
Nivolumab (Anti-PD-1 Ab)	Melanoma	Melanoma
	non-small cell lung cancer	non-small cell lung cancer
	renal cell carcinoma	renal cell carcinoma
	Hodgkin's lymphoma	Hodgkin's lymphoma
	Head neck cancer	Head neck cancer
	MSI-H/dMMR colorectal cancer	gastric cancer
	hepatocellular carcinoma	diffuse malignant pleural mesothelioma
	small cell lung cancer	Esophageal cancer
		MSI-high colorectal cancer

Pembrolizumab (Anti-PD-1 Ab)	Melanoma	Melanoma
	non-small cell lung cancer	non-small cell lung cancer
	Head neck cancer	Urothelial cancer
	Hodgkin's lymphoma	MSI-high solid tumor
	Urothelial cancer	renal cell carcinoma ^∗^ (combination)
	MSI-high colorectal cancer	Head neck cancer ^∗∗^ (mono/combination)
	MSI-high cancer	
	gastric cancer	
	cervical cancer	
	hepatocellular carcinoma	
	Merkel cell carcinoma	
	renal cell carcinoma	
	endometrial cancer	

Avelmab (Anti-PD-L1 Ab)	Merkel cell carcinoma	Merkel cell carcinoma
	renal cell carcinoma	renal cell carcinoma ^∗^ (combination)
	Urothelial cancer	

Atezolizumab (Anti-PD-L1 Ab)	Urothelial cancer	non-small cell lung cancer
	non-small cell lung cancer	extensive-disease small cell lung cancer.
	breast cencer	triple negative breast cancer
	small cell lung cancer	

Durvalumab (Anti-PD-L1 Ab)	Urothelial cancer	non-small cell lung cancer (stage 3)
	non-small cell lung cancer	

Ipilimumab (Anti-CTLA4 Ab)	Melanoma	Melanoma ^∗∗∗^ (mono/combination)
	renal cell carcinoma	renal cell carcinoma ^∗∗∗∗^ (combination)
	MSI-H/dMMR colorectal cancer	

Kymriah (CAR-T)	B-ALL (<25 yars-old)	B-ALL (<25 yars-old)
	DLBCL (Hodgkin's lymphoma)	

Yescarta (CAR-T)	DLBCL (Hodgkin's lymphoma)	not approved

Sipuleucel-T (Provenge) (DC-vaccine)	Prostate cancer	not approved

^∗^Combination with axitinib, ^∗∗^monotherapy or combination with chemotherapy, ^∗∗∗^monotherapy or combination with nivolumab, ^∗∗∗∗^combination with nivolumab. MSI-H/dMMR: microsatellite instability-high/deficient mismatch repair; B-ALL: B cell acute lymphoblastic leukemia; DLBCL: diffuse large B cell lymphoma; Ab: antibody; PD-1: programmed death-1; PD-L1: programmed death ligand-1; CAR: chimeric antigen receptor; DC: dendritic cell; FDA: Food and Drug Administration; EMA: European Medicines Agency; MHLW: Ministry of Health, Labour and Welfare.
